# Icosahedral virus structures and the protein data bank

**DOI:** 10.1016/j.jbc.2021.100554

**Published:** 2021-03-17

**Authors:** John E. Johnson, Arthur J. Olson

**Affiliations:** Department of Integrative Structural and Computational Biology, The Scripps Research Institute, La Jolla, California, USA

**Keywords:** virus structure, virus assembly, structural model, structure–function, virology, HRV, Human Rhinovirus, MCP, major capsid protein, NCS, noncrystallographic symmetry, PDB, Protein Data Bank, PV, Poliovirus, SBMV, Southern Bean Mosaic Virus, TBSV, Tomato Bushy Stunt Virus, VIPER, virus particle explorer

## Abstract

The structural study of icosahedral viruses has a long and impactful history in both crystallographic methodology and molecular biology. The evolution of the Protein Data Bank has paralleled and supported these studies providing readily accessible formats dealing with novel features associated with viral particle symmetries and subunit interactions. This overview describes the growth in size and complexity of icosahedral viruses from the first early studies of small RNA plant viruses and human picornaviruses up to the larger and more complex bacterial phage, insect, and human disease viruses such as Zika, hepatitis B, Adeno and Polyoma virus. The analysis of icosahedral viral capsid protein domain folds has shown striking similarities, with the beta jelly roll motif observed across multiple evolutionarily divergent species. The icosahedral symmetry of viruses drove the development of noncrystallographic symmetry averaging as a powerful phasing method, and the constraints of maintaining this symmetry resulted in the concept of quasi-equivalence in viral structures. Symmetry also played an important early role in demonstrating the power of cryo-electron microscopy as an alternative to crystallography in generating atomic resolution structures of these viruses. The Protein Data Bank has been a critical resource for assembling and disseminating these structures to a wide community, and the virus particle explorer (VIPER) was developed to enable users to easily generate and view complete viral capsid structures from their asymmetric building blocks. Finally, we share a personal perspective on the early use of computer graphics to communicate the intricacies, interactions, and beauty of these virus structures.

The Protein Data Bank (PDB) has played a pivotal role for disseminating virus structure atomic coordinates in a manner that has been useful for virologists and other noncrystallographers in recent years. Achieving this utility has had a number of iterations with a variety of contributions from structural virologists and the developers of graphics programs. Indeed, there has been a refreshing level of cooperation among a number of participants that has brought the enterprise to its current state of effectiveness. In this contribution the early developments of virus crystallography are briefly presented, the progress in the ease of use of the PDB data resource for virologists is described, then the history of some features of structural virology as documented in icosahedral virus PDB submissions since 1984.

## Early years

### Icosahedral virus structure—a short history

The structural study of icosahedral viruses has a long and impactful history in propelling forward both crystallographic methodology and the understanding of molecular biology. In the 1930s, Bernal and Fankuchen made the first crystallographic studies of a spherical plant virus, Tomato Bushy Stunt Virus (TBSV) (1). At that time, they were able to determine the particle size from the lattice layer lines and conclude that “a body-centered cell is probable as it accords with the dodecahedral habit by Fedorov's law.” In 1956, Crick and Watson reasoned that the total mass of a protein shell the size of TBSV would necessitate many identical units to accommodate the limited ribonucleic acid capacity inside the shell (2). That same year Caspar’s diffraction studies of TBSV demonstrated that the particle itself had crystallographic cubic symmetry, consistent with an icosahedral geometry (3). Since the icosahedron has 60 symmetrically identical positions, there must be 60 identical building blocks in the virus. In the 1960s, Caspar and Klug noted that since TBSV had more than 60 chemically identical subunits in its capsid, the same chemical entity (protein subunit) must be accommodated in more than one distinct neighborhood. Thus, they developed the concept of “quasi-symmetry” that hypothesized flexible accommodations of the protein subunits and used a “T-number” (as devised by Buckminister Fuller for his icosahedral domes) to characterize the number of quasi-symmetric protein units in the icosahedral asymmetric unit (4). They could not, at the time, establish the structural nature of the quasi-symmetry.

**Figure 1:**
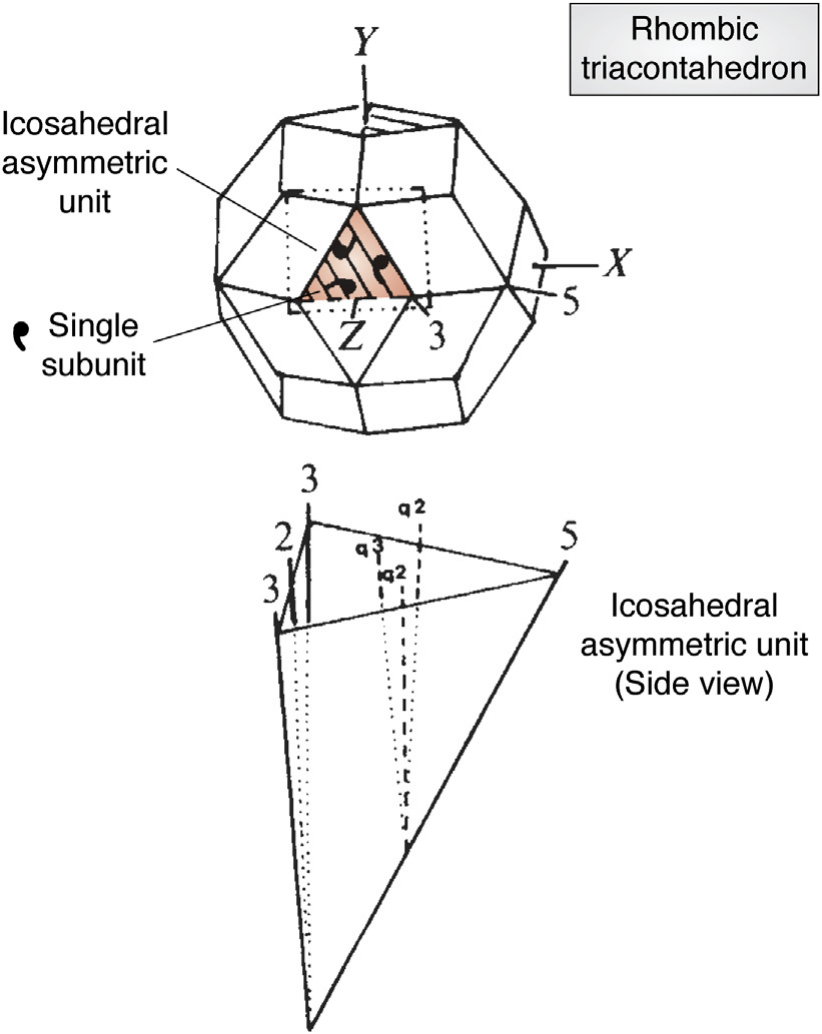
***Top*, a rhombic triacontahedron depicting the T = 3 quasi-symmetric quaternary structure of Tomato Bushy Stunt Virus and Southern Bean Mosaic Virus, the first two icosahedral virus structures deposited in the PDB.** The *triangle* enclosing the “commas” is the icosahedral asymmetric unit and a “comma” represents a single subunit with the folds shown in Figure 2. They are related by an approximate q3 fold axis at the center of the triangle. *Bottom*, a side view of the icosahedral asymmetric unit with the particle center at the *bottom* of the figure where the boundary icosahedral symmetry elements (5-3-3-5) intersect. Quasi-symmetry elements are defined by vectors representing q2 and q3. Note, unlike the icosahedral symmetry elements, they do not intersect at the center.

It wasn’t until the late 1970s that computers and crystallographic computing methods enabled atomic-level resolution of structures as large as intact virus particles. The 5.5 Å resolution crystal structure of TBSV, determined in 1977 in the Harrison Lab (5), was enabled by fivefold molecular averaging, utilizing Bricogne’s newly developed noncrystallographic symmetry (NCS) method. The structure revealed a rhombic triacontahedral viral capsid with 180 protein subunits with two domains, a shell and projecting domain with similar topological folds connected by a linker polypeptide. The structure showed variabilities between subunit dimers surrounding the five (quasi -2fold) and twofold icosahedral axes, in both the curvature between shell domains and orientation of the projecting dimer domains (Fig. 1). The subsequent 2.9 Å resolution structure of TBSV from the same lab elucidated the atomic nature of the quasi-equivalence in this virus structure. It revealed ordered polypeptide strands in the interfaces between the dimers around twofold axes that were absent at the dimers around the fivefold axes, with the three ordered strands forming a beta-barrel structure at the icosahedral threefold axes. This structure showed that the quasi-equivalent “accommodations” postulated by Caspar and Klug entailed significant morphological and topological modifications.

### First icosahedral virus coordinate deposits to the pdb

**Figure 2:**
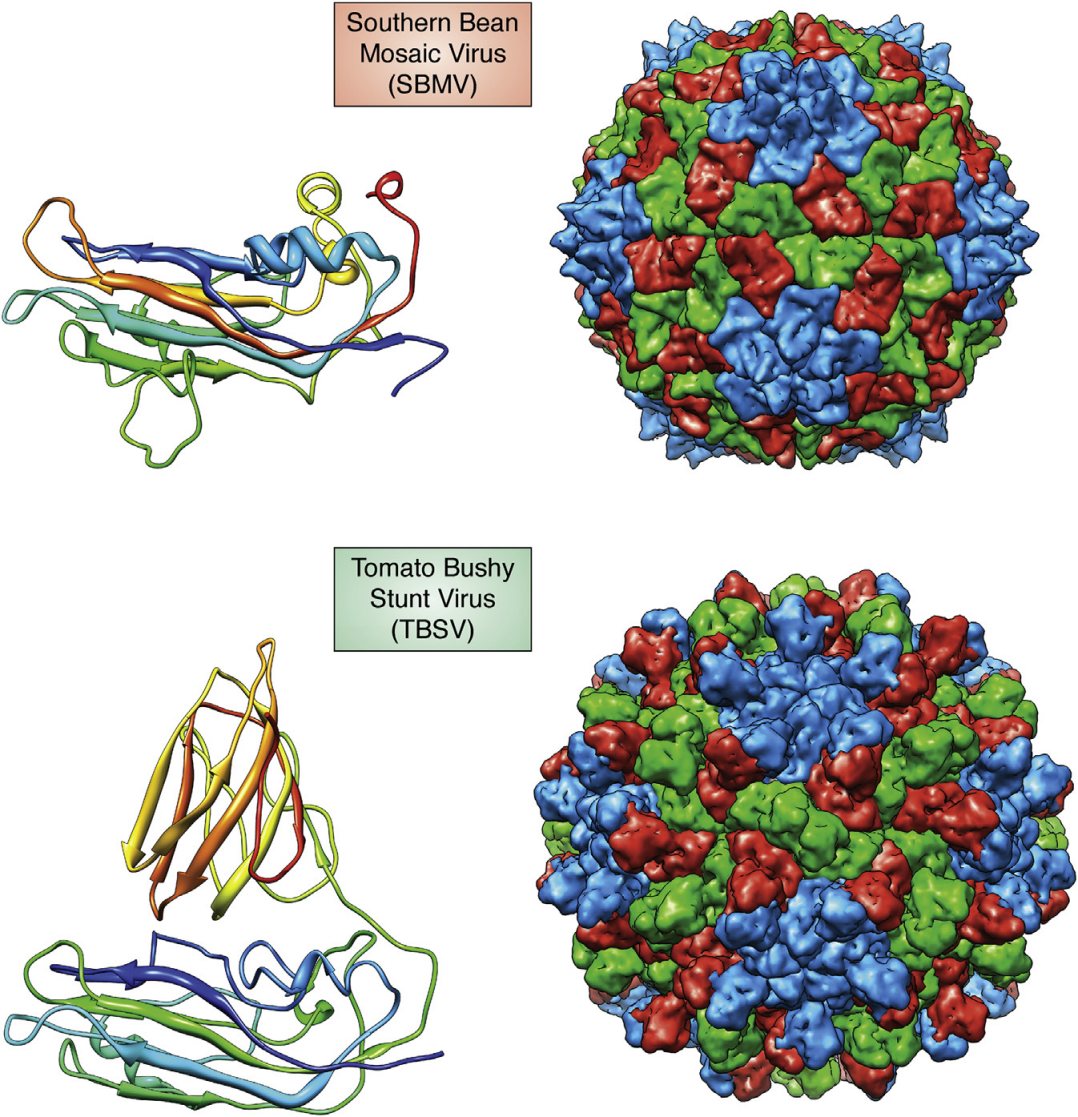
**Southern Bean Mosaic Virus (SBMV) (*top*) and Tomato Bushy Stunt Virus (TBSV) (*bottom*) were the first icosahedral virus structure coordinates deposited to the PDB in 1984.** TBSV was determined in 1978 (6) and SBMV in 1980 (7). Unexpectedly the shell forming domains of both the virus subunits were closely similar and formed what is now known as the virus jellyroll topology. TBSV has an additional C-terminal domain that forms prominent dimers on the capsid surface.

TBSV (6) from the Harrison lab and Southern Bean Mosaic Virus (SBMV) in 1980 (7) from the Rossmann Lab were the first virus structures deposited in the PDB. Both of these RNA plant viruses have T = 3 quasi symmetry (4) The close similarity in the shell forming domain of the protein subunits in these two viruses was unexpected, but immediately noticed by Rossmann and Andrew Leslie from the chain tracing of the polypeptides in the electron density plotted on plexiglass sheets. The fold (Fig. 2) is now called the virus jelly roll topology (8). The atomic models of both TBSV and SBMV protein subunits (like all protein models at that time) were constructed with a Richards’ Box (9), an invention of Fred Richards of Yale that allowed the simultaneous viewing of electron density and the associated physical brass model employing a half-silvered mirror. The primary model builders of TBSV were Harrison and one of us (AJO) and the SBMV models were mainly built by Rossmann and Cele Abad-Zapatero. The physical models were translated into 3d coordinates by yardstick measurements to a plumb bob positioned at each atom position. As was common in the early days of the PDB, there was a significant delay of the deposition of these structures; the TBSV coordinates were released in July 1984 (2tbv) and the SBMV coordinates in October 1984 (3sbv; subsequently updated with 4sbv). At this time there were 175 total entries in the PDB. The deposition process was at one level straightforward since the coordinates to be deposited were those found in the crystallographic asymmetric units. TBSV crystallized in a body-centered cubic space group with the particle center at a ***32*** symmetry site, so all but the 5fold icosahedral axes were part of the crystal lattice. A pentamer of trimers (icosahedral asymmetric units) were deposited. SBMV crystallized in a rhombohedral space group with the particle center at a ***23*** symmetry site implying 2 pentamers of trimers in the crystallographic asymmetric unit and 30 subunit coordinate sets deposited. Since NCS was critical in phase determination and improvement, it was noteworthy that TBSV had fivefold NCS and SBMV tenfold NCS. This specified that the SBMV crystal form had 1.4 times the phase improvement power of the TBSV crystal form since phase enhancement goes as the square root of the NCS.

Both structure determinations required numerous person-years of effort (TBSV; 1968–1978, SBMV; 1972–1980) and in the end there were two Nature papers (6, 7) with state-of-the-art figures for virologists to ponder. The reaction to the work was respectful, but the vast majority of virologists had no idea how to use this information. The problem was manifold; first, outside of the crystallography community, there were few investigators that knew what to do with a set of coordinates and there were no widely available computer graphics instruments or programs except for those that would plot coordinates on a 2d plotter (10); second, even crystallographers encountered a new problem more challenging than the simpler point group symmetries confronted with enzymes and other proteins; generating the biologically relevant structure from the deposited coordinates. Addressing the latter was challenging. Given the crystallographic asymmetric unit of coordinates, how do you create the full virus particle? The TBSV coordinates represented 1/12 of the particle and SBMV represented 1/6 of the particle. Operating on the deposited coordinates with the appropriate crystallographic symmetry operators to generate an intact particle was challenging even for crystallographers (including those that deposited the coordinates!). As a result, the first structures did not have an immediate impact for those that were not well versed in the matrices of the icosahedral point group.

Translating virus coordinates into useful 3d graphics was first accomplished by one of us (AJO) with TBSV along with other efforts in the use computer graphics to generate 3d models of viruses. A narrative history of the development of the first 3d tour of a virus particle as well as other virus animations is provided in Box 1. It has been exciting to see how these efforts have been mirrored by what is now possible for individuals on a desktop computer decades later. The movie provided a readily understandable description of the importance of the pioneering virus structures and their relevance for virology.

Box 1 **Personal reflections on computer graphics and animation of virus structures by Arthur J. Olson**

**Table:** 

The Harrison Lab acquired an Evans & Southerland (E&S) Multi Picture System (MPS) in 1978. This new technology embodied a monochrome vector-based display capable of addressing the equivalent of 8000X8000 pixels and a 3D graphics processor that enabled interactive manipulations of complex 3D structures. Because at the time there were no suitable molecular modeling programs, by substituting molecules for buildings, I hacked a demo Fortran program from E &S called “Architecture,” which enabled manipulation of wireframe models of buildings. I could then read in the molecular coordinates of the three distinct subunits of Tomato Bushy Stunt Virus (TBSV) as well as produce geometric icosahedral models. We were then able to see and interact with a complete virion at atomic resolution and get a new perspective on the nature of quasi-equivalence, as postulated by Caspar and Klug (4). I was enamored with the new device and wanted to show a wider audience what we had seen. Since we had no way of capturing our interactions with the structure, we borrowed reel-to-reel video tape recorder and camera and shot some ad hoc manipulations. It was hardly a great success since we could only capture live interaction. I only showed it a couple of times outside of Harrison’s Harvard lab.
In September of 1979, I moved from my post-doc position in the Harrison Lab to Lawrence Berkeley Lab to join the newly formed National Resource for Computation in Chemistry (NRCC). I joined as the Staff Scientist in charge of crystallographic computing. I was able to hire a talented post-doc, TJ O’Donnell, who was also very interested in computer graphics, having worked on creating the Death Star for the first Star Wars movie. I was able to acquire the same model E&S system that I had been using at Harvard. Together TJ and I designed and developed a general-purpose interactive graphics interpreter in Fortran that we called GRAMPS (Graphics for the Multi-Picture System). In its design, we included a number of animation capabilities, as well as enabling the types of symmetry manipulations that would be useful for molecular complexes. Key to the utility of GRAMPS was the ability to assign objects to input devices (like the multi-dial box that came with the system) and transform objects interactively) (76).
At the top of my mind was the making of a computer-generated animation explaining the TBSV structure by showing the viral capsid atomic structure, its symmetry, and configurational changes. With GRAMPS, this became possible. I bought a Bolex 16 mm movie camera and wired its single-frame mechanism to the keyboard terminal bell, which we could activate through a GRAMPS command.
Because the software language and manual manipulations were interactively interpreted, an animation script could be written as it was being developed, and the timing of events could be adjusted interactively. Once a script was completed, it could be played back on the E&S display and filmed frame by frame. Using this approach, the resulting TBSV 16 mm film was one continuous scene with no film edits. The first recorded version was monochrome with white vector lines on a phosphor screen. Subsequently we were able to borrow a new color MPS from Evans and Southerland and run the script again, with color commands inserted, which made a significant difference in distinguishing the three distinct subunits.
The animated 16 mm film called “TBSV,” produced at the NRCC, was widely seen in the 1980s into 1990s. It was first shown at the Association for Computing Machinery (ACM) Siggraph Conference in 1981 to a computer graphics crowd. I had added an accompanying piano soundtrack of Scott Joplin Rags (Maple Leaf Rag and The Entertainer) as a nod to the early silent movies. “TBSV” along with my contemporaneous CG animation “ADAM-a Dial Activated Man” got a very positive response. Subsequently showing the film around on talks about the virus structure, I started to get a number of requests for copies, and so started distributing the 16 mm copies at cost. That dropped off as 16 mm movie projectors became rare and was eventually substituted with an analogue video version, distributed on VHS videotape.
The 8-min film itself was one of the first of its kind, especially for molecular animation. It was the ideal medium at the time for explaining the complex and dynamic nature of viral capsids. It tells a story as a progression from the fundamental icosahedral symmetry of the capsid to the nature of quasi-symmetry, showing conformational changes between the distinct subunits, in assembly and swelling. The E&S graphics processor was the most powerful at the time, but it could not display the entire atomic scale capsid, with its millions of vectors, at interactive rates. In animation mode, however, I was able to capture a complete pentameric subassembly, with our 16 mm animation camera. As mentioned above, the entire film is a single continuous shot, which keeps the viewer in context as the content and scale of the images change. With the subsequent publication of two more virus structures, Cow Pea Mosaic Virus (CPMV) and Satellite Tobacco Mosaic Virus (STNV), I produced another 16 mm film called “Virus Wars,” which compared the three structures. In 1986, in collaboration with Jim Hogle, then at Scripps, we made 16 mm animation of his structure of the poliovirus, with Jim’s narration.
In 1983, TBSV, the virus made another appearance on film. This time it was in an OmniMax Film produced for the Horizons Pavilion at the Disney EPCOT Center and shown in dome projection. I created the animation for the segment on the color E&S system that I had purchased after moving to Scripps in late 1981. In collaboration with Nelson Max, the vector drawings were distorted so that they looked correct projected onto the Omnimax dome (77). The data generated from the GRAMPS script was written on 60 reels of 9in 6250bpi magnetic tape and mailed to a laser writer in Minneapolis to be written onto 70 mm filmstock using three 70 mm frames per image.
In the early 2000s, I had a digital copies of the original 16 mm films made. The digital versions of “TBSV” (https://www.youtube.com/watch?v=FhLAC_Ol8_0), “Virus Wars” (https://youtu.be/D0REwUXu50I) and “Poliovirus” (https://www.youtube.com/watch?v=JV2gte4o-d4&t=6s) have now been made available on YouTube.

### Human icosahedral virus structures

The ssRNA picornaviruses, Poliovirus (PV) (11) and Human Rhinovirus (HRV) (12), were reported at 2.9 Å resolution in 1985. The years between the plant virus structures and these animal virus structures saw remarkable advances in the availability of synchrotron X-ray radiation, computer graphics, and high-performance supercomputers. Access to these technologies reduced the time required to determine virus structures as well as dramatically improving the ease of modeling protein structures and refining the coordinates. These virus models were of good quality and of high impact for virology. They provided important insights for virus evolution, immunology, and pathology and lead to mechanistic understanding of a class of antiviral agents. The PV and HRV structures were closely similar to each other with so-called pseudo T = 3 symmetry. This referred to the fact that they had structures that were immediately reminiscent of TBSV and SBMV with three viral jelly roles in the icosahedral asymmetric unit. In contrast to the previous structures, each jelly roll had a different sequence, thus there were three different, but structurally similar, proteins in the icosahedral asymmetric unit. This leads directly to the hypothesis that the picornaviruses were related to the plant viruses with a gene triplication and subsequent independent evolution of the plant virus subunit gene, a proposal strengthened by the subsequent discovery of picornavirus-like plant virus structures (13). There existed a plethora of molecular immunological data for both picornaviruses, and this was rapidly related to the structures (14, 15) to identify the neutralization immunogen (NIM) regions of the capsid that were closely similar for both viruses. The structures also suggested the location of the receptor binding site on the capsid as there was a deep depression near the fivefold axes referred to as the “canyon” that seemed like a logical place for the receptor to make a strong interaction with the particle (16). Finally, the structures revealed the binding site of a known antiviral agent to picornaviruses (17) leading to an extensive structure-based drug design enterprise (18). The first version of the HRV coordinates (2hrv) was released by the pdb in July 1986 (subsequently replaced by 4hrv) and the PV coordinates (2plv) were released in October 1989.

### Virus particle explorer

**Figure 3:**
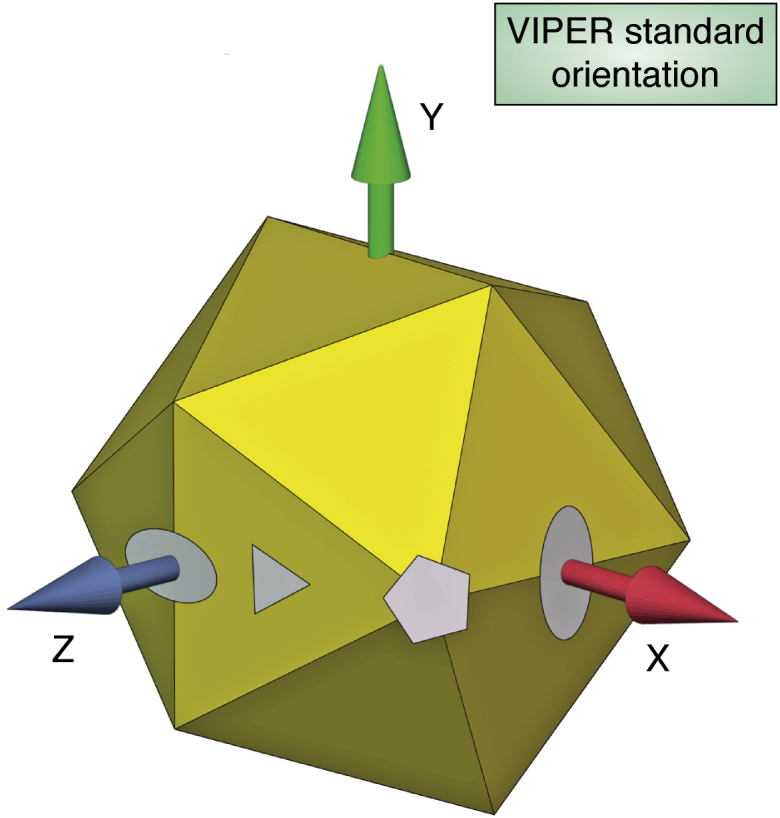
**The standard orientation of an icosahedron in an orthogonal coordinate system defined by Vijay Reddy as the “VIPER orientation.”** When the crystallographic coordinates of an icosahedral virus particle were deposited to the PDB, he converted them to this orientation allowing the straightforward expansion to the full icosahedron through selection of the 60 resident matrices for the icosahedral point group (23). The conventions developed in VIPER were adapted by the Protein Data Bank, allowing ready access to any oligomer of the subunits (28).

Generally speaking, virologists were not able to use the coordinates for their research because of the difficult learning curves associated with the early graphics programs and the complications associated with icosahedral symmetry operators. A notable early exception was the Institute for Virology at the University of Wisconsin, Madison where Jean Yves Sgro embraced computer graphics as a postdoctoral associate and facilitated a variety of informative and insightful illustrations of nodaviruses (19), parvoviruses (20), and picornaviruses (21). These efforts directly impacted the research of virologists Collin Parrish, Paul Kaesberg, Roland Rueckert, and Ann Palmenberg and began a dialog between virologists and crystallographers regarding information that the coordinates could provide. Input from Sgro and others guided efforts at Purdue to develop straightforward lists of residues at subunit interfaces and residues solvent-exposed on the interior and exterior surfaces. These were extended to determine the character of subunit interfaces by developing contact tables where comparisons could be made between quasi-equivalent interfaces and comparable icosahedral interfaces and the character of the stabilizing forces determined (*i.e.*, ionic, hydrophobic, etc.) (22). These were assembled by virus crystallographers at Purdue for coordinates in the pdb and supplied to virologists to develop structure-based investigations of virus biology. Initially this was done on an ad hoc basis, but it became clear that there would be a great benefit to do this in a systematic manner for all virus coordinates as they became available. This was the start of an icosahedral virus coordinate database known as VIrus Particle ExploreR or VIPER that has been led since its inception in 1994 by Vijay Reddy with multiple releases over the years (23, 24, 25, 26, 27). Reddy developed principles that were eventually formalized, and these allowed the entire process from coordinate deposition to creating a VIPER database entry to be automated. A key step facilitated the procedure. The coordinates were converted to what is known as the VIPER standard orientation in an orthogonal coordinate system as shown in Figure 3. Once the coordinates for the crystallographic asymmetric unit are in that orientation, it is straightforward to generate a full virus particle or any oligomeric unit with the 60 resident matrices for the icosahedral point group. Each VIPER entry provides a summary of the biophysical characteristics of the particle as well as a variety of ready-made, publication quality graphics. VIPER is a heavily used facility with ∼4000 sessions/month that include some type of download or utility use and its publications have received over 500 citations. The VIPER standards have been incorporated into the PDB and all of the virus coordinates have been remediated to allow straightforward generation of biologically relevant oligomers (28).

### Virus crystallography comes of age

**Figure 4:**
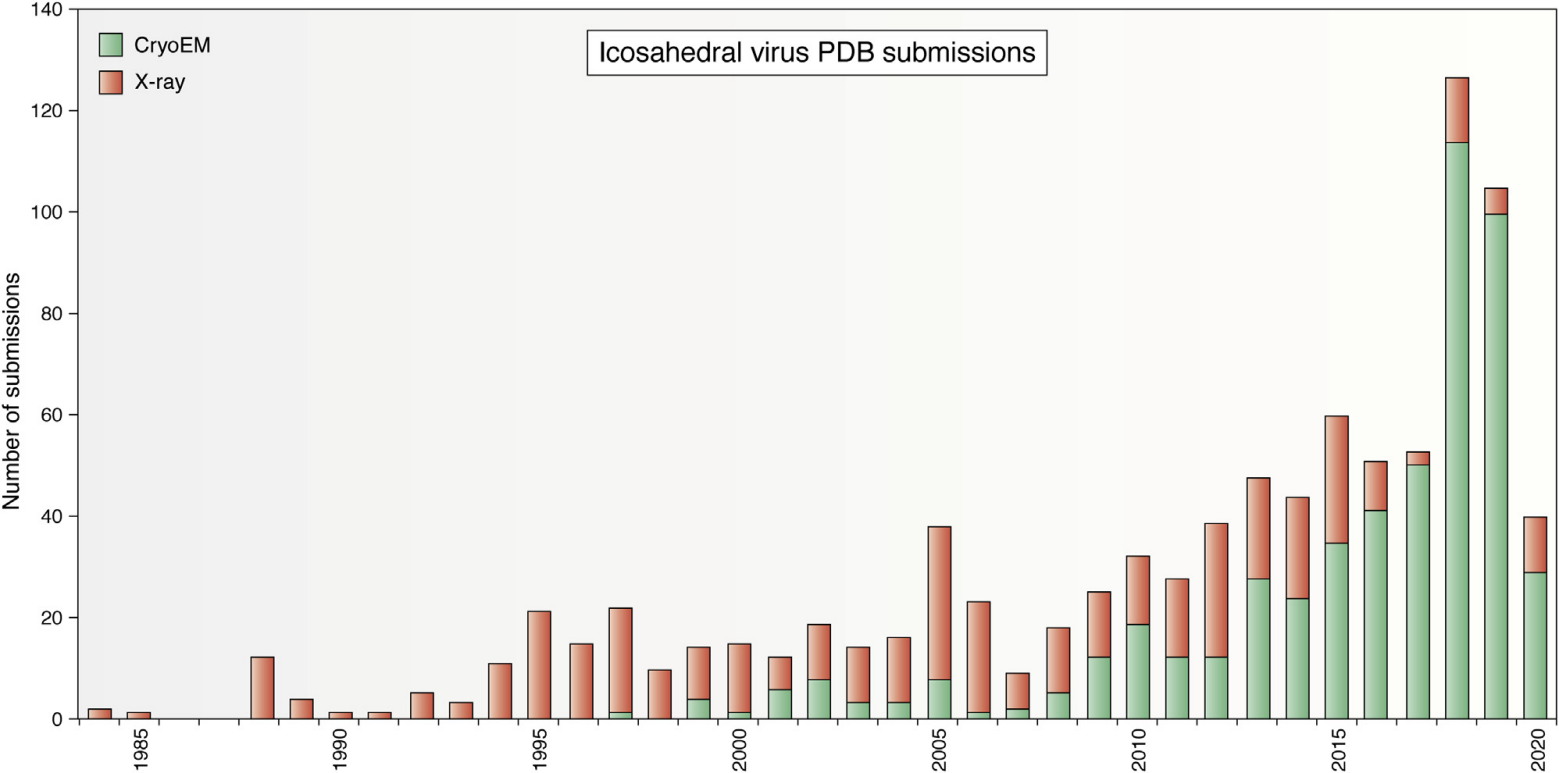
**A histogram showing the number of icosahedral virus structures deposited to the PDB each year and the method of structure determination (X-ray crystallography or cryo-electron microscopy).** Since 2010, cryoEM has become the dominant method of structure determination.

Following the first four virus structures deposited to the PDB, the pace picked up (Fig. 4). By 1990, there were 18 entries, including 11 variations of HRV14 determined with different mutations and drugs bound to an internal pocket, two additional unique picornavirus structures, and a plant picorna-like virus. The latter showed, for the first time, discernible fragments of the RNA genome within the capsid implying that portions of the RNA had the symmetry of the protein capsid (13). A striking feature of the structures deposited to this point was their uniformity; they were all ∼30 nM diameter formed by 180 subunits that all displayed the jellyroll fold.

A variety of technology advances resulted in 59 virus entries by 1995. The first ssDNA virus, the bacteriophage phi X 174, was submitted in 1991 and displayed the jellyroll fold in the two different major gene products that formed the icosahedral asymmetric unit of the T = 1 particle (29). The mammalian, ssDNA, canine parvovirus, had a single subunit type with a jellyroll fold that was highly elaborated with extended loops that formed a T = 1 particle (30). Black beetle virus was the first RNA insect virus submitted to the PDB, and it displayed a structure remarkably similar to SBMV, but had an interesting autocatalytic maturation step that was well characterized in the structure (31). Significantly the first virus with a subunit that did not have a jellyroll fold, the RNA bacteriophage MS2, was submitted in this time frame (32) as well as dsDNA tumor viruses with T = 7l surface lattices that did not have the anticipated distribution of hexamers predicted by quasi-equivalence theory; the enigmatic simian virus 40 (33) and polyomavirus (34). Surprisingly the latter viruses also had the jellyroll fold in their subunits. Thus, through 1995 subunits with the jellyroll were found in all but one virus submitted, and the jellyroll spanned viruses with RNA and DNA genomes and those infecting bacteria, plants, insects, and mammals! Virus structures were becoming rather monotonous.

The count of icosahedral virus particle deposits was at 134 by 2000 and some viruses of exceptional importance were deposited between 1995 and 2000. The transcriptionally active Blue Tongue Virus core was among those with the greatest impact. This dsRNA virus in the family reovirus has a T = 13 l outer shell formed by a two-domain protein with a helical interior and a protruding viral jellyroll on the outside. Interior and adjacent to the RNA was a T = 1 core formed by 120 copies of a predominantly helical protein with a totally different fold from other viral capsid proteins. The deposition in 1998 set a number of records for a virus. At 700 Å in diameter it was the largest virus in the PDB; it had the largest T number at 13; and it was the first to have shells with two different symmetries (35). Two years later the coordinates of the core of reovirus (36) were submitted to the PDB allowing comparisons of orbiviridae and reoviridae in the family reovirus. Insights were developing into the evolution of picornaviruses from plant viruses as structures were reported of putative intermediates in the process (37). The first high-resolution procapsid structure was reported in 1997 for Phi X 174 (38), a particle on the maturation pathway to the infectious virus that included scaffolding proteins that guide the assembly of more complex viruses and are then released and are not present in the mature particle. The majority of other viruses submitted in this time frame were picornaviruses, the vast majority infecting humans with many having mutations with interesting phenotypes and coordinates associated with structure-based drug design. The coordinates of an insect picornavirus, with a drastically different genome organization from human picornaviruses, were submitted in 1998, but the structure was recognizably similar to human picornaviruses albeit without the canyon and NIM sites (39). Notably the first coordinates employing cryoEM density were submitted in this time frame, but they were based on the fit of known, homologous, structures into low-resolution cryoEM density. They were important, however, in mapping Fab fragments attached to picornaviruses (40) as well as receptor–picornavirus interactions (41, 42).

Approximately 100 icosahedral virus structures were submitted to the PDB between 2001 and 2005. A quarter of these were structures based in part on cryoEM density. The “gold standard” in this time frame for cryoEM was sub-nanometer resolution structures, although many were at much lower resolution. The cryoEM density together with X-ray-based coordinates of individual capsid proteins provided the first detailed models of flavi (43) and alpha viruses (44). These are 600 Å, ssRNA, icosahedral, membrane-containing particles that were refractory to high-resolution crystallography. These structures were high impact as the viruses cause widespread morbidity and mortality throughout the world. Although the models were pseudo atomic, later high-resolution structures showed them to be remarkably accurate. Flavi and alphaviruses contain glycoproteins with a characteristic fold (45, 46) that is different from the viral jellyroll and that clearly relates these viruses at a subunit structural level. Their capsid architectures are, however, strikingly different with flaviviruses displaying a capsid with 180 subunits, which are not consistent with T = 3 quasi-equivalence, and alphaviruses showing a classic T = 4 quasi-equivalent capsid formed with two different, but structurally similar, glycoproteins. A second area that gained exceptional momentum in this time frame were structural studies by X-ray crystallography and cryoEM of dsDNA bacteriophage particles. An atomic model of the T = 7*l* Hong Kong 97 VLP (47) demonstrated a new capsid protein fold (HK97-fold) that is now recognized as the canonical capsid protein fold for tailed, dsDNA bacteriophages and clearly relates the mammalian herpesviruses to tailed, dsDNA phage (48). The first detailed models of these complex, dsDNA phage particles were also reported in this period for Phi 29 (49), PRD1 (50), and T4 (51). In this time frame and with high-resolution capsid protein structures, it was clear that the dsDNA phage divided into two groups, the tailed phage with the HK97 fold in their subunits and the membrane containing (*e.g.*, PRD1) that had a subunit fold similar to the mammalian adenoviruses. The PDB had become an exceptionally valuable resource for understanding virus particle evolution.

Another 100 icosahedral virus structures were deposited from 2006 to 2010. The novel focus in this time frame was the maturation of virus particles, the study of membrane containing viruses, and the denovo structure determination of virus particles by cryoEM. Bacteriophage maturation was exemplified by studies of HK97 VLPs where X-ray crystallography and cryoEM identified subtleties of protein subunit reorganization (52, 53) and subunit refolding (54). A new level of understanding of large membrane-containing phage in the PRD1-adenovirus lineage was achieved with the moderate resolution X-ray structure of PM2 (55). CryoEM structures were now determined in some cases with no reference to X-ray subunit models with dsRNA cytoplasmic polyhedrosis virus determined at 3.9 Å (56) and dsDNA, tailed bacteriophage Epsilon15 determined at 4.5 Å resolution (57). The latter two structures had pushed EM to its limits with the existing CCD detector technology. The future of cryoEM was in the making during this time frame with a publication describing the direct electron detector (58), an instrument that would revolutionize structural virology and all of structural biology during the next 5 years.

The years 2011 to 2015 saw 200 icosahedral virus submissions to the PDB with more than half using cryoEM for either *de novo* structure determination or combined with X-ray models of particle components to generate pseudo-atomic models. The time frame of 2012 saw many coordinate deposits employing cryoEM at sub 5 Å resolution with *de novo* structure determination. Many virus structures determined with either X-ray or cryoEM were modified versions of previously deposited coordinates such as Adeno-Associated Virus that had a changed receptor target for gene therapy (59) or picornaviruses that were inactivated during vaccine development (60). Among the largest structures deposited to the PDB to that point was the 75mD sulfolobus turreted icosahedral virus (STIV) purified from the archea, sulfolobus, in the hot springs of Yellowstone National Park. The structure was determined with a 4.5 Å cryoEM map that allowed *de novo* tracing of the major capsid protein (MCP) and fitting of X-ray structures of two other capsid gene products (61). Dengue virus structures of both immature and mature particles were reported at 4.5 Å and 4.1 Å respectively (62). Multiple expansion intermediates of the T7 bacteriophage were reported with one cryoEM structure at better than 4 Å and the infectious virus at 3.5 Å (63)

A total of 907 icosahedral virus model coordinates were submitted to the PDB by July 2020, an increase of 356 between 2016 and 2020. Only 41 of these were determined by X-ray crystallography. The dominance of cryoEM for icosahedral virus structure determination is nearly complete. In 2013 one of us (JEJ) wrote an autobiographical review titled “Confessions of an icosahedral virus crystallographer” (64). His “confession,” following a personal history of virus crystallography and the description of the cryoEM structure of STIV that was determined at 4.5 Å resolution, was that he no longer did icosahedral virus crystallography, only cryoEM. It is interesting that the real moment of transition occurred in 2010 when the structure of adenovirus was determined by X-ray crystallography (65) and cryoEM (66) and published together in the journal Science. A news and views was supplied by Steve Harrison (67) and his opinion was that the cryoEM density was of higher quality. The final revised coordinates for the cryoEM model were deposited in 2017 (68) and the X-ray model in 2018 (69). Among other notable entries in this period was the sub 2 Å cryoEM structure of an adeno-associated virus particle that took into account a variety of technical details to achieve 1.8 Å resolution (70).

## Conclusions

**Figure 5:**
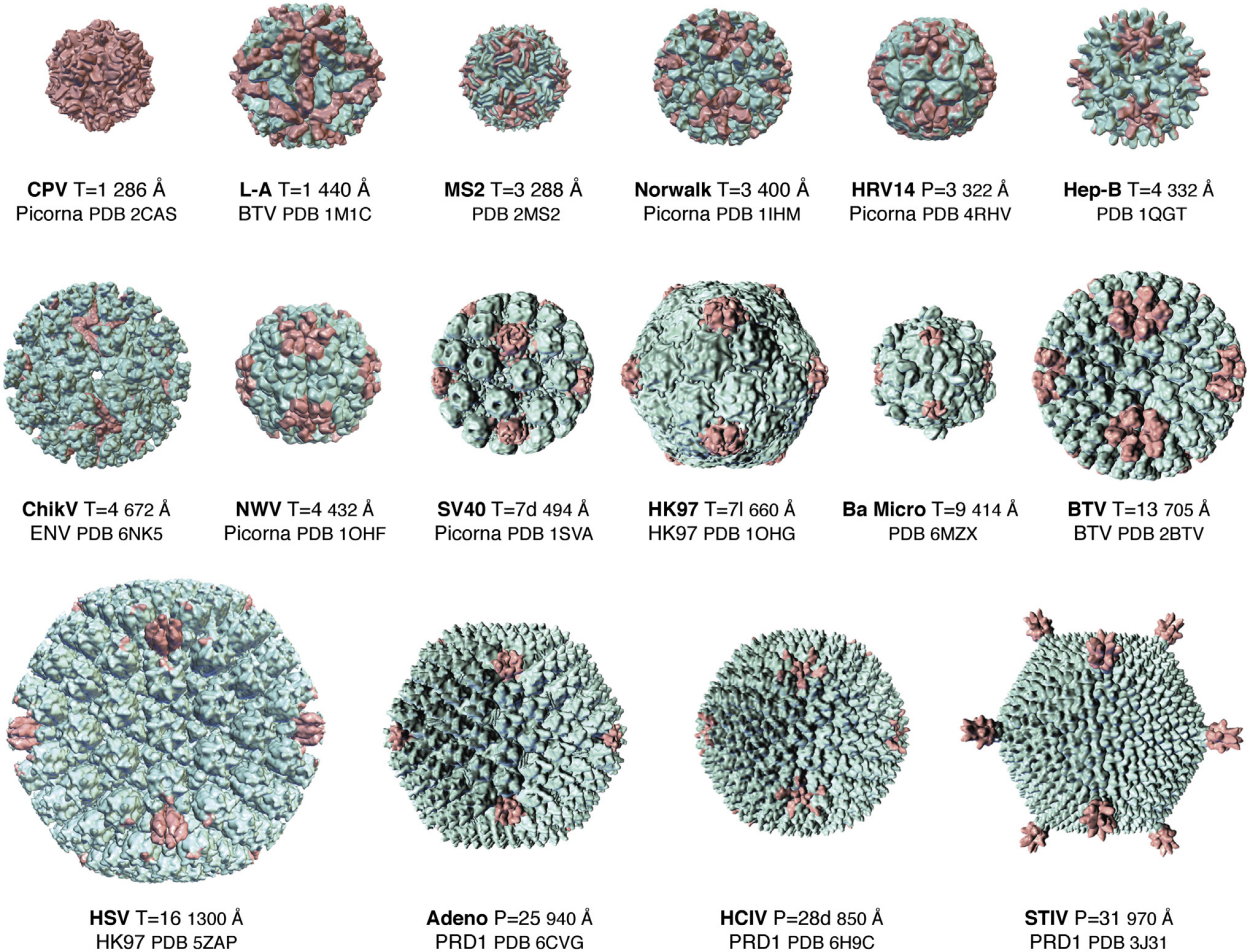
**A selection of virus structures from the PDB, demonstrating the size range and quasi-equivalence of particles determined to near-atomic resolution.** For each virus there is a name abbreviation, the quasi or pseudo equivalence of the capsid, the particle diameter, the pdb identifier for the coordinates, and the lineage based on the major capsid protein subunit structure; Picorna-like. Blue Tongue Virus (BTV)-like, HK97-like, PRD1-like as described by Abrescia *et al.* (75). Row1 Canine Parvo Virus, L-A virus, MS2, Norwalk Virus. Row 2 Human Rhinovirus 14, Hepatitis B virus, Chikungunya Virus, Nudaurelia Omega Capensis Virus. Row 3 Simian Virus 40, Hong Kong 97, Bacterial Microcompartment shell assembly, Blue Tongue Virus. Row 4 Herpes Simplex Virus, Adenovirus, Haloarcula californiae icosahedral virus, Sulfolobus turreted icosahedral virus. Figures were produced with PMV (Python Molecular Viewer) (78).

**Figure 6:**
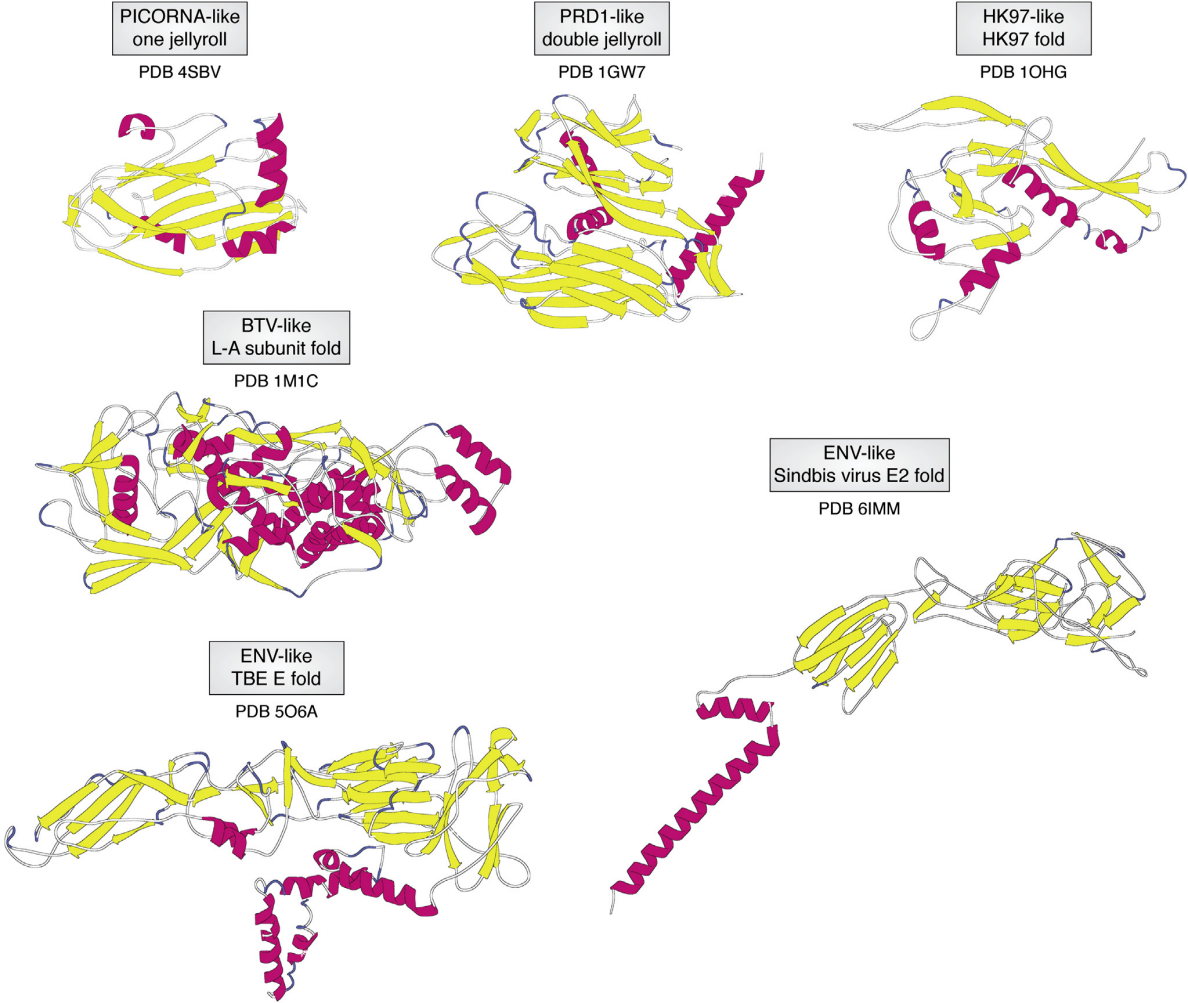
**The folds employed to define the lineages of capsids in Abrescia *et al.*** (75)**.** For each subunit fold, the lineage is given as well as the PDB identifier for the coordinates used to generate the specific fold. The folds for the enveloped alpha and flavi viruses are from Sindbis Virus and Tick-Borne Encephalitis Virus, respectively. Figures were produced with PMV (Python Molecular Viewer) (78).

The virus structures deposited to the PDB over the last 40 years have provided fundamental insights into virus assembly and maturation (71), the roles of the capsid in virus lifecycles (72) had a major impact on the development of particle-based antiviral agents (73), the immunology of virus particles (74), and structure-based virus classification, contributing to proposals for virus evolution (75). A summary of structure-based virus classification is a fitting conclusion to this article. Based on MCP structures, Abrescia *et al.* (75) list four icosahedral viral lineages that they refer to as picorna-like (ssRNA and DNA viruses characterized by MCPs with a single jellyroll), PRD1-like (dsDNA viruses characterized by MCPs containing the double jellyroll fold), Blue Tongue virus-like (dsRNA viruses characterized by a T = 1 nucleoprotein particle with a dominantly alpha helical fold and, usually, an outer shell formed by proteins that often include a jellyroll), and HK97-like (dsDNA viruses infecting bacteria and also herpesviridae). In addition to these lineages, there are the icosahedral enveloped lineages (ENV) comprised of alpha and flavi-viruses that are ssRNA and contain surface glyco-proteins that are fusogenic and/or attachment. The flavi-virus glycoprotein is referred to as the E-protein and the alpha virus proteins E1 and E2. Figure 5 illustrates a variety of virus structures in the pdb with their identifier, diameter, and lineage. Figure 6 shows the tertiary structure folds of MCPs that form the capsids or glycoprotein surface of the ENV lineage.

There can be little doubt that the PDB, together with innovations incorporated from the Virus Particle Explorer database, has played a pivotal role in making icosahedral virus structures accessible for the plethora of studies that have placed many aspects of virology on a structural foundation.

## Conflict of interest

The authors declare that they have no conflicts of interest with the contents of this article.
